# Anti‐U1‐RNP‐Positive Inflammatory Myopathy Presenting With Dysphagia: A Case Report

**DOI:** 10.1002/ccr3.73676

**Published:** 2026-10-05

**Authors:** Anjlee, Sana Rasheed, Moosa Ashfaq, Simran Bai, Ali Jan Yaqubi, Ahmed Asad Raza

**Affiliations:** ^1^ Department of Medicine and Surgery Ghulam Muhammad Mahar Medical College Sukkur Pakistan; ^2^ Department of Medicine and Surgery Jinnah Sindh Medical University Karachi Pakistan; ^3^ Department of General Medicine Jinnah Postgraduate Medical Center Karachi Pakistan; ^4^ Department of Medicine Kabul University of Medical Sciences Abu Ali Sina Kabul Afghanistan

**Keywords:** anti‐U1‐RNP antibodies, autoimmune myositis, dysphagia, inflammatory myopathy, mixed connective tissue disease, polymyositis

## Abstract

High‐titer anti‐U1‐RNP antibodies may present with myopathy without fulfilling MCTD criteria. We report a 38‐year‐old woman with proximal weakness, elevated muscle enzymes, anti‐U1‐RNP positivity, and inflammatory myopathy on EMG/MRI, but lacking classic overlap features. Early recognition enabled immunosuppressive treatment and prevention of aspiration‐related complications.

## Key Clinical Message

Clinicians should suspect anti‐U1‐RNP‐associated autoimmune myopathy in patients with progressive proximal weakness and dysphagia, even without classic mixed connective tissue disease features. Markedly elevated muscle enzymes and high‐titer anti‐U1‐RNP antibodies warrant evaluation. Early immunosuppressive therapy is essential to prevent severe complications, including aspiration pneumonia.

## 1 Introduction

Mixed Connective Tissue Disease (MCTD) is a systemic autoimmune disorder first described by Sharp et al. in 1972, characterized by overlapping clinical features of systemic lupus erythematosus (SLE), polymyositis (PM), and systemic sclerosis (SSc), with a defining serological hallmark of high‐titer anti‐U1 ribonucleoprotein (U1‐RNP) antibodies [1]. The estimated prevalence of MCTD is approximately 3–37 per 100,000 individuals, with a strong female predominance [2].

Musculoskeletal involvement, including proximal muscle weakness and myositis, occurs in approximately 25%–75% of MCTD patients and may be the dominant presenting feature [3]. Dysphagia secondary to pharyngeal muscle involvement, though well‐recognized in polymyositis and dermatomyositis, is less frequently reported as a prominent early feature of MCTD and represents a clinically challenging and potentially life‐threatening complication due to aspiration risk [4].

Delayed diagnosis of MCTD is common, particularly when myositis is the predominant feature and skin or joint manifestations are absent. Elevated muscle enzymes combined with positive anti‐U1‐RNP antibodies and characteristic electromyographic and imaging findings are critical for accurate diagnosis [5]. The combination of a markedly prolonged, myopathy‐dominant disease course culminating in the late and abrupt onset of life‐threatening pharyngeal dysphagia is distinctly uncommon and constitutes the principal educational message of the present report. We report a case of a 38‐year‐old female who presented with a 6‐year history of progressive proximal muscle weakness and acute onset of oropharyngeal dysphagia, in whom investigation revealed a high‐titer anti‐U1‐RNP‐positive inflammatory myopathy.

## 2 Case Presentation

A 38‐year‐old female patient, with no significant past medical history, no history of consanguinity, and no prior miscarriages, was admitted with a chief complaint of generalized weakness predominantly in the upper limbs for 6 years and dysphagia of one and a half weeks duration. There was no history of diarrhea, constipation, gastrointestinal bleeding (hematemesis, hematochezia, or melena), hematuria, or lower respiratory tract symptoms. The patient reported on‐and‐off low‐grade fever that responded to antipyretic medication, with no identifiable aggravating features.

The weakness had been progressive over 6 years. Initially, the patient was unable to resist objects placed in the upper extremities; however, by the time of admission, she was unable to lift her legs from the bed against gravity. The patient had previously been seen by general practitioners for this weakness and had been found to have anemia on complete blood count (CBC), for which she received iron supplementation. Despite supplementation, the anemia persisted, raising suspicion for an underlying systemic cause. The dysphagia was initially to solids but rapidly progressed to liquids over one and a half weeks, involving the third (pharyngeal) phase of swallowing. This prompted referral from a dermatologist to the internal medicine department. The patient had no known family history of autoimmune disorders or neuromuscular diseases. She is a housemaker, and her quality of life had significantly declined over the 6‐year period due to her inability to perform basic motor tasks and the psychological distress caused by the sudden onset of dysphagia. The chronological sequence of clinical events is summarized in Table 1.

**TABLE 1:** Timeline of clinical events.

Time point	Clinical events
6 years prior to admission	Gradual onset of proximal muscle weakness (upper limbs initially)
Over 6 years	Progressive worsening of weakness, eventually involving lower limbs
Prior evaluations	Diagnosed with anemia; received iron supplementation without improvement
1.5 weeks prior to admission	Onset of dysphagia (initially solids, rapidly progressing to liquids)
Day of admission	Clinical evaluation, physical examination, and laboratory investigations performed
Hospital stay (Day 1–3)	Elevated CPK and aldolase; positive ANA and anti‐U1‐RNP antibodies; EMG and MRI confirmed inflammatory myopathy
Day 1–3 of treatment	Initiation of IV methylprednisolone (500 mg daily for 3 days)
Day 4	Significant improvement in dysphagia and muscle strength
Post‐pulse therapy	Transition to oral prednisolone (1 mg/kg/day)
During hospitalization	Methotrexate initiated as steroid‐sparing agent
At discharge	Patient clinically improved; advised follow‐up and monitoring
Follow‐up (to be added)	Clinical and biochemical monitoring (CPK, strength, swallowing function)

### 2.1 Physical Examination

On examination, the patient was conscious and oriented. Cranial nerve examination (CNX) was intact. Muscle power, assessed in the proximal muscle groups, was graded 4/5 in the bilateral proximal upper limbs (deltoid and biceps) and 2/5 in the bilateral proximal lower limbs (hip flexors and quadriceps), the patient being unable to lift the legs against gravity; distal muscle strength was preserved. No skin rash, Gottron's papules, heliotrope rash, or mechanic's hands were identified on dermatological inspection. There were no joint effusions or synovitis on musculoskeletal examination. Respiratory, cardiovascular, and abdominal examinations were unremarkable. The distribution and severity of muscle weakness, together with bulbar involvement, are graded serially in Table 2.

**TABLE 2:** Longitudinal muscle strength (Medical Research Council [MRC] grade), serum creatine kinase, dysphagia status, and treatment course.

Parameter	At admission (Day 0)	After pulse therapy (Day 4)	At 4‐week follow‐up
Proximal upper limbs (deltoid, biceps), MRC grade	4/5	5/5	5/5
Proximal lower limbs (hip flexors, quadriceps), MRC grade	2/5 (unable to lift legs against gravity)	4/5	4–5/5 (sustained)
Distal limb muscles	Preserved	Preserved	Preserved
Bulbar/pharyngeal (swallowing)	Severe oropharyngeal dysphagia (solids progressing to liquids)	Marked improvement; safe oral intake restored	No dysphagia; normal oral intake
Cranial nerves (I–XII)	Intact	Intact	Intact
Serum creatine kinase (IU/L)	9883	Not re‐measured	560
Treatment	IV methylprednisolone 500 mg/day for 3 days	Oral prednisolone 1 mg/kg/day; methotrexate + folic acid initiated	Prednisolone taper; methotrexate maintenance

### 2.2 Investigations

A comprehensive workup was undertaken. Laboratory investigations and their results are summarized in Table 3.

**TABLE 3:** Summary of laboratory investigations.

Investigation	Result	Reference range	Interpretation
Hemoglobin	9.3 g/dL	11.0–14.5 g/dL	Low—anemia
Hematocrit	32.0%	34.5%–45.4%	Low
MCV	75 fL	78.1–95.3 fL	Microcytic
MCH	22 pg	25.3–31.7 pg	Hypochromic
RDW	17%	12.1%–16.9%	Elevated
WBC	8.1 × 10^9^/L	4.6–10.8 × 10^9^/L	Normal
ESR (Westergren)	67 mm/h	0–20 mm/h	Elevated
CRP	116 mg/L	0–14 mg/L	Markedly elevated
Serum CPK	9883 IU/L	34–145 IU/L	Massively elevated—active myositis
Serum Aldolase	75.6 U/L	2.5–10 U/L	Markedly elevated
Serum Creatinine	0.3 mg/dL	0.6–1.1 mg/dL	Normal renal function
eGFR	> 60 mL/min/1.73 m^2^	≥ 60	Normal
ANA (cytoplasmic)	Positive (speckled)	Negative	Positive
U1‐RNP antibodies	17.67 U/mL	> 5.0 Positive	Strongly Positive
SS‐A/Ro antibodies	3.57 U/mL	> 5.0 Positive	Equivocal
SS‐B/La antibodies	0.75 U/mL	> 12.5 Positive	Negative
Sm antibodies	1.05 U/mL	> 5.0 Positive	Negative
Scl‐70 antibodies	0.91 U/mL	> 5.0 Positive	Negative
Anti‐dsDNA (IgG)	37.14 IU/mL	> 46 Positive	Equivocal (35–46)
Serum C3	1.81	Normal range	Normal
Serum C4	0.24	Normal range	Normal
Direct Coombs Test	Positive (1+)	Negative	Positive—immune haemolysis

The peripheral blood film demonstrated anisocytosis, hypochromic microcytic red cells, poikilocytosis, polychromasia, and elliptical cells, consistent with iron deficiency anemia; the persistence of anemia despite supplementation also raised the possibility of anemia of chronic disease. A positive direct Coombs test (1+) was noted; however, no additional laboratory evidence of active hemolysis was documented, and this finding was therefore of uncertain significance (see Discussion).

The serum ENA (Extractable Nuclear Antigen) profile revealed a strongly positive U1‐RNP antibody level of 17.67 U/mL (reference: > 5.0 U/mL = positive), with equivocal SS‐A/Ro and negative SS‐B/La, Sm, and Scl‐70 antibodies. Anti‐dsDNA (IgG) was equivocal at 37.14 IU/mL (equivocal range: 35–46 IU/mL). Serum complement levels (C3 and C4) were within normal limits.

### 2.3 Electromyography and Nerve Conduction Studies

Nerve conduction studies (NCS) of the right median (APB) and posterior tibial (AH) motor nerves and right median (F2) and sural antidromic sensory nerves were all within normal limits, effectively excluding a neuropathic etiology. Needle electromyography (EMG) demonstrated small, short‐duration motor unit potentials with early full recruitment across all tested muscles (right tibialis anterior, gastrocnemius, vastus medialis, iliopsoas, flexor digitorum, brachioradialis, biceps, deltoid, trapezius, and upper and lower thoracic paraspinals), consistent with an acute to subacute generalized non‐irritable myopathy. The electrodiagnostic conclusion included metabolic myopathy and burnt‐out inflammatory myositis as probable differential diagnoses.

### 2.4 Magnetic Resonance Imaging

MRI of the left thigh (plain, performed on 10 March 2026) using T1, T2, STIR, and PD fat‐sat sequences demonstrated diffuse hyperintense signal within the proximal thigh and quadriceps muscles bilaterally on T2‐weighted and STIR images, consistent with muscle edema and inflammation. Mild joint effusion was noted within the visualized knee joint. There was no evidence of abnormal femoral bone marrow signal, fracture, bony erosion, or lymphadenopathy. The neurovascular bundle appeared normal. The radiological conclusion suggested inflammatory myositis as the primary diagnosis, with early muscular dystrophy as an alternative consideration, with recommendations for NCS/EMG correlation and rheumatology consultation.

### 2.5 Management and Clinical Course

Given the severity of proximal muscle weakness, markedly elevated muscle enzymes, and rapidly progressive pharyngeal dysphagia with significant aspiration risk, the patient was initiated on high‐dose intravenous corticosteroid therapy. She received pulse methylprednisolone (Solu‐Medrol) 500 mg intravenously once daily for three consecutive days.

A marked clinical response was observed following pulse therapy. The patient's dysphagia improved significantly, with restoration of safe oral intake. Muscle strength improved notably: upper limb power increased to 5/5 bilaterally, while lower limb strength improved to 4/5 on the Medical Research Council (MRC) scale.

Following pulse therapy, the patient was transitioned to oral corticosteroids (prednisolone 1 mg/kg/day) as maintenance therapy, with a plan for gradual tapering based on clinical response and serial monitoring of muscle enzyme levels.

Methotrexate was initiated as a steroid‐sparing immunosuppressive agent, alongside folic acid supplementation. Supportive therapy included analgesics for myalgia and multivitamins. Swallowing precautions were advised, and the patient was counseled regarding aspiration risk.

### 2.6 Follow‐Up and Outcomes

The patient was discharged in a stable condition with detailed instructions for regular follow‐up in the rheumatology clinic. A structured follow‐up plan was established to ensure close monitoring of disease activity and treatment response. This included serial measurement of serum creatine phosphokinase (CPK) levels, complete blood counts, and liver function tests for ongoing methotrexate surveillance, along with periodic clinical assessment of proximal muscle strength, functional status, and swallowing ability to monitor for recurrence of dysphagia or progression of myopathy.

At the 4‐week follow‐up visit, the patient demonstrated sustained and clinically meaningful improvement in muscle strength, with no new functional limitations reported. Serum CPK levels showed a significant downward trend, decreasing to 560 IU/L. This value was approaching the normal reference range for most laboratories, typically approximately 34–135 IU/L (may vary slightly depending on the assay and laboratory standards). The normalization trend was consistent with biochemical remission of muscle inflammation.

Importantly, no relapse of dysphagia was observed, and the patient was able to maintain adequate oral intake without difficulty. Overall, both clinical and biochemical parameters indicated a favorable response to therapy, with continued stability under close rheumatological follow‐up. The longitudinal clinical, biochemical, and treatment course is summarized in Table 2.

## 3 Discussion

This case presents a 38‐year‐old female with a high‐titer anti‐U1‐RNP‐positive inflammatory myopathy with pharyngeal dysphagia. The clinical picture comprised progressive proximal muscle weakness, dysphagia, and high‐titer anti‐U1‐RNP antibodies, together with markedly elevated muscle enzymes, a characteristic myopathic EMG pattern, and MRI evidence of muscle inflammation.

Ever since MCTD has been established, its diagnostic criteria and classification systems have demonstrated variability. Four are the most widely recognized: Sharp [6], Alarcón‐Segovia and Villareal [7], Kasukawa and Miyawaki [8] and Kahn and Appeboom [9]. Despite these differing methodologies, a high titer of anti‐U1‐RNP antibodies remains a fundamental requirement across all systems. To further complicate clinical assessment, cases have been reported where the classic MCTD symptomatology was present but with minimal or no anti‐U1‐RNP antibodies, as well as individuals with a markedly high titers of anti‐U1‐RNP antibodies in the absence of classic manifestations of MCTD [10]. Owing to this heterogenous and evolving clinical presentation, MCTD is frequently misdiagnosed or subject to delayed diagnosis.

Conventionally MCTD is defined serologically by the presence of high‐titer anti‐U1‐RNP antibodies, which bind the U1 small nuclear ribonucleoprotein complex and mediate autoimmune tissue damage. In this patient, the high‐titer anti‐U1‐RNP antibody (17.67 U/mL) represented the principal serological hallmark and diagnostic anchor for MCTD, whereas the accompanying ANA positivity with a cytoplasmic speckled pattern was a supportive but non‐specific finding [11].

An explicit appraisal against the established classification systems is warranted before this presentation is labeled MCTD. A high‐titer anti‐U1‐RNP antibody is the obligatory serological requirement shared by the Sharp, Alarcón‐Segovia, Kasukawa, and Kahn criteria [6, 7, 8, 9], and this patient clearly satisfied that requirement. However, each of these systems additionally requires one or more classic overlap manifestations. The Alarcón‐Segovia criteria require at least three of five clinical features (hand edema, synovitis, myositis, Raynaud phenomenon, and acrosclerosis), whereas this patient exhibited only myositis; the Kasukawa criteria require a common symptom of Raynaud phenomenon or swollen fingers/hands, both of which were absent; the Kahn criteria require Raynaud phenomenon together with at least two of swollen fingers, synovitis, or myositis; and the Sharp criteria require a combination of major features extending beyond myositis and serology alone [6, 7, 8, 9]. On formal assessment, therefore, this patient did not fulfill any of the four established classification criteria for MCTD, because the requisite overlap features—Raynaud phenomenon, swollen hands, and sclerodactyly—were absent at the time of evaluation. Accordingly, the presentation is most accurately designated an anti‐U1‐RNP‐positive inflammatory myopathy rather than definite MCTD. High‐titer anti‐U1‐RNP antibodies in a myositis‐predominant phenotype are nonetheless increasingly recognized as a distinct subset that may represent an early, incomplete, or myositis‐dominant point on the MCTD spectrum, and a proportion of such patients evolve to fulfill complete criteria over time [11, 12, 13]. This diagnostic ambiguity is itself instructive, underscoring the need for longitudinal serological and clinical surveillance, since overlap features may emerge during follow‐up and reclassify the disease as established MCTD.

The markedly elevated CPK (9883 IU/L, approximately 68 times the upper limit of normal) and aldolase (75.6 U/L) indicated severe active skeletal muscle injury, and the subsequent decline in CPK to 560 IU/L mirrored the clinical response to immunosuppression [14]. Although serum creatine kinase is a useful biomarker for monitoring disease activity, it does not always parallel muscle strength or clinical severity [14, 15]. The non‐irritable myopathic EMG pattern, characterized by the absence of fibrillations and positive sharp waves, is more typical of a chronic or ‘burnt‐out’ myopathy than of florid active myositis; in this patient it most plausibly reflected the prolonged 6‐year course, upon which the markedly raised muscle enzymes indicated superimposed, biochemically active muscle injury [14].

MCTD associated myositis mimics idiopathic inflammatory myopathies both clinically and histologically, which often leads to diagnostic confusion. Histological studies have demonstrated heterogeneous results of myopathology of MCTD associated myositis showing polymyositis and dermatomyositis type findings [16, 17]. Patients diagnosed with idiopathic inflammatory myopathies, which show poor therapeutic response or demonstrate atypical features, should be evaluated for myositis associated antibodies, particularly anti‐U1 RNP antibodies, which can help reclassify the disease to MCTD [12].

The oropharyngeal dysphagia in this patient, involving the pharyngeal phase of swallowing and progressing from solids to liquids within a week and a half, represents involvement of the striated pharyngeal musculature. Pharyngeal dysphagia in inflammatory myopathy occurs due to inflammation of the cricopharyngeal and inferior pharyngeal constrictor muscles, and is a recognized but underreported feature of MCTD with myositis overlap [4]. Its rapid progression in this case underscores the clinical urgency and risk of aspiration pneumonia. Pharyngeal dysphagia is recognized as an adverse prognostic marker in inflammatory myopathy, being associated with a higher risk of aspiration, malnutrition, and death, and therefore warrants prompt recognition and aggressive immunosuppression [18].

Several features distinguish the present report from previously published cases of MCTD‐associated inflammatory myopathy. In the clinicopathological case series reported by Sakamoto et al., MCTD‐related myositis typically followed a subacute course and was recognized within months of symptom onset [17]. Similarly, in the cohort of anti‐U1‐RNP‐positive myositis patients characterized by Casal‐Domínguez et al., muscle involvement was usually identified during active multisystem disease, and dysphagia, although described, was seldom the presenting emergency [12]. Esophageal and pharyngeal involvement in MCTD has likewise been reported predominantly as a chronic or subclinical finding rather than as an acute, rapidly progressive complaint [4]. Against this background, two features render the present case distinctive and educationally valuable: first, the exceptionally prolonged six‐year, myopathy‐dominant course that preceded serological diagnosis; and second, the abrupt evolution of severe oropharyngeal dysphagia over approximately 10 days as the event that finally precipitated diagnosis. This trajectory underscores the importance of considering an autoimmune myopathy in any patient with longstanding, unexplained proximal weakness, even in the absence of the classic cutaneous or vascular stigmata of connective tissue disease.

Anemia seen in MCTD is often multifactorial and can easily be misattributed to iron deficiency or chronic disease type. Hematological abnormalities such as anemia and leukopenia are reportedly observed in 30%–40% of MCTD patients [2, 13, 19]. Of significance to our case, Coombs positive hemolytic anemia has also been described in the past while even rarer manifestations such as pure red cell aplasia further expand the hematological spectrum. A high index of suspicion should be maintained in cases of refractory or unexplained anemia for an underlying autoimmune process and appropriate evaluation should be pursued [20, 21, 22].

The positive direct Coombs test (1+) in this patient should be interpreted with caution. A positive direct antiglobulin test alone is neither specific for systemic autoimmunity nor sufficient to establish autoimmune hemolytic anemia (AIHA), and no additional laboratory evidence of active hemolysis (such as reticulocytosis, an elevated lactate dehydrogenase, raised unconjugated bilirubin, or reduced haptoglobin) was documented. Although AIHA has been reported in MCTD and anti‐U1‐RNP‐associated disease and may contribute to anemia in some patients [23], the isolated low‐titer positive Coombs test here is best regarded as a non‐specific finding of uncertain significance rather than definitive evidence of a broader autoimmune diathesis. The equivocal anti‐dsDNA and normal complement levels argue against florid SLE overlap but do not exclude it. Taken together with the refractory microcytic anemia—which in retrospect represented a missed opportunity for earlier systemic evaluation—these findings were consistent with the decision to pursue a structured autoimmune workup, and serial monitoring for evolving hemolysis is warranted [20, 21, 22, 23].

The absence of skin manifestations such as heliotrope rash or Gottron's papules differentiates this presentation from dermatomyositis; however, the strong myositis phenotype, the U1‐RNP serology, and the absence of significant interstitial lung disease or scleroderma features are consistent with an anti‐U1‐RNP‐positive inflammatory myopathy on the MCTD spectrum rather than isolated polymyositis or dermatomyositis [3].

The 6‐year diagnostic delay in this case is consistent with the known challenge of recognizing anti‐U1‐RNP‐associated myopathy and MCTD‐spectrum disease, particularly when myopathy is the dominant feature and serological workup is not initially pursued. This case emphasizes the importance of including autoimmune myositis panel and anti‐ENA serology in any patient presenting with proximal muscle weakness and anemia refractory to supplementation.

## 4 Limitations

Despite the clinical significance of this case, several limitations must be acknowledged. First, a muscle biopsy was not performed, which precluded histopathological confirmation of the specific myositis subtype (e.g., polymyositis vs. dermatomyositis‐like patterns). Second, the “burnt‐out” non‐irritable myopathic pattern observed on needle electromyography (EMG) may be a consequence of the prolonged six‐year diagnostic delay rather than a reflection of the acute disease state. Third, while the clinical response to therapy was robust, the improvement in pharyngeal dysphagia was assessed primarily through clinical observation and the restoration of oral intake rather than objective instrumental measures, such as a video‐fluoroscopic swallow study. Additionally, although an extractable nuclear antigen (ENA) panel was completed, a broader myositis‐specific antibody panel was not conducted, which might have provided further phenotypic characterization of the myopathy. Finally, as a single case report, these findings regarding the presentation and treatment of anti‐U1‐RNP‐positive inflammatory myopathy with severe dysphagia may not be generalized to all patient populations.

## 5 Patient's Perspective

I experienced significant difficulty in performing my daily activities due to gradually worsening weakness, and I felt distressed when I suddenly developed difficulty swallowing. After receiving treatment, I noticed a marked improvement in my strength and was able to eat normally again, which greatly improved my quality of life. I feel satisfied with the treatment and relieved to finally have a clear diagnosis after years of symptoms.

## 6 Conclusion

This case illustrates a high‐titer anti‐U1‐RNP‐positive inflammatory myopathy with acute pharyngeal dysphagia as the precipitating complaint leading to diagnosis. Although the patient did not fulfill the established classification criteria for MCTD owing to the absence of classic overlap features, the constellation of markedly elevated muscle enzymes, high‐titer anti‐U1‐RNP antibodies, a non‐irritable myopathic EMG pattern, and MRI‐confirmed muscle inflammation defined a clinically significant autoimmune myopathy that may represent an early or myositis‐dominant presentation on the MCTD spectrum. Timely recognition of anti‐U1‐RNP‐associated myopathy is critical to initiate immunosuppressive therapy and prevent life‐threatening complications. Clinicians should maintain a high index of suspicion for autoimmune myopathy in patients with progressive proximal weakness and unexplained persistent anemia, and a comprehensive autoimmune panel including anti‐ENA and anti‐U1‐RNP antibodies should be obtained early in the diagnostic workup.

## Author Contributions

**Anjlee:** conceptualization, writing – original draft, writing – review and editing, supervision, validation. **Sana Rasheed:** writing – review and editing, writing – original draft. **Moosa Ashfaq:** writing – original draft, writing – review and editing. **Simran Bai:** writing – original draft, writing – review and editing. **Ali Jan Yaqubi:** writing – original draft, writing – review and editing. **Ahmed Asad Raza:** writing – original draft, writing – review and editing, supervision, visualization.

## Funding

The authors have nothing to report.

## Ethics Statement

Our institution does not require ethical approval for reporting individual case reports.

## Consent

Written informed consent from the patient was obtained according to journal guidelines.

## Conflicts of Interest

The authors declare no conflicts of interest.

## Data Availability

Data sharing not applicable to this article as no datasets were generated or analyzed during the current study.
